# A novel approach on designing ultrahigh burnup metallic TWR fuels: Upsetting the current technological limits

**DOI:** 10.1557/s43577-022-00420-4

**Published:** 2022-11-03

**Authors:** Linna Feng, Yuwen Xu, Jie Qiu, Xiang Liu, Chunyang Wen, Zhengyu Qian, Wenbo Liu, Wei Yan, Yanfen Li, Zhaohao Wang, Shilun Zheng, Shaoqiang Guo, Tan Shi, Chenyang Lu, Junli Gou, Liangxing Li, Jianqiang Shan, James F. Stubbins, Long Gu, Di Yun

**Affiliations:** 1 School of Nuclear Science and Technology, Xi’an Jiaotong University, Xi’an, China; 2 Nuclear Power Institute of China, Chengdu, China; 3 Laboratory for Advanced Nuclear Energy Theory and Applications, Zhejiang Institute of Modern Physics, Department of Physics, Zhejiang University, Hangzhou, China; 4 CAS Key Laboratory of Nuclear Materials and Safety Assessment, Institute of Metal Research, Chinese Academy of Sciences, Shenyang, China; 5 Shi-Changxu Innovation Center for Advanced Materials, Institute of Metal Research, Chinese Academy of Sciences, Shenyang, China; 6 Department of Nuclear, Plasma and Radiological Engineering, University of Illinois at Urbana-Champaign, Champaign, USA; 7 Institute of Modern Physics, Chinese Academy of Sciences, Lanzhou, China; 8 University of Chinese Academy of Sciences, Beijing, China; 9 School of Nuclear Science and Technology, Lanzhou University, Lanzhou, China

**Keywords:** Zero carbon, Traveling wave reactor, Nuclear fuels, Irradiation effects, High burnup

## Abstract

**Abstract:**

The grand challenge of “net-zero carbon” emission calls for technological breakthroughs in energy production. The traveling wave reactor (TWR) is designed to provide economical and safe nuclear power and solve imminent problems, including limited uranium resources and radiotoxicity burdens from back-end fuel reprocessing/disposal. However, qualification of fuels and materials for TWR remains challenging and it sets an “end of the road” mark on the route of R&D of this technology. In this article, a novel approach is proposed to maneuver reactor operations and utilize high-temperature transients to mitigate the challenges raised by envisioned TWR service environment. Annular U-50Zr fuel and oxidation dispersion strengthened (ODS) steels are proposed to be used instead of the current U-10Zr and HT-9 ferritic/martensitic steels. In addition, irradiation-accelerated transport of Mn and Cr to the cladding surface to form a protective oxide layer as a self-repairing mechanism was discovered and is believed capable of mitigating long-term corrosion. This work represents an attempt to disruptively overcome current technological limits in the TWR fuels.

**Impact statement:**

After the Fukushima accident in 2011, the entire nuclear industry calls for a major technological breakthrough that addresses the following three fundamental issues: (1) Reducing spent nuclear fuel reprocessing demands, (2) reducing the probability of a severe accident, and (3) reducing the energy production cost per kilowatt-hour. An inherently safe and ultralong life fast neutron reactor fuel form can be such one stone that kills the three birds. In light of the recent development findings on U-50Zr fuels, we hereby propose a disruptive, conceptual metallic fuel design that can serve the following purposes at the same time: (1) Reaching ultrahigh burnup of above 40% FIMA, (2) possessing strong inherent safety features, and (3) extending current limits on fast neutron irradiation dose to be far beyond 200 dpa. We believe that this technology will be able to bring about revolutionary changes to the nuclear industry by significantly lowering the operational costs as well as improving the reactor system safety to a large extent.

**Graphical abstract:**

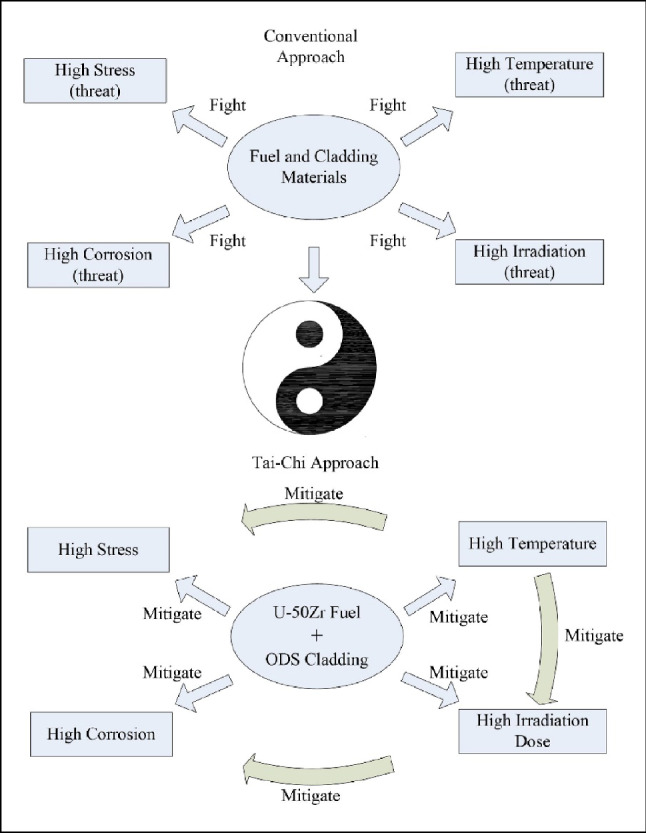

**Supplementary information:**

The online version contains supplementary material available at 10.1557/s43577-022-00420-4.

## Background

After the COVID-19 outbreak, climate catastrophe will be the next crisis faced by humanity. Stopping global warming requires significant reductions in the emission of greenhouse gases into the atmosphere. Electricity production accounts for about 27% of the greenhouse gas emissions originating from human activities, but it represents a solution scheme that is of ample importance.^1^ Nuclear energy is a viable clean energy source that can supply power continuously with minimum geographical limitations, and no other clean energy source is comparable to nuclear energy in terms of power density and capacity factor. Without nuclear power, the cost of achieving “zero carbon” electricity is impractically high and it is difficult to move forward with decarburization.

The traveling wave reactor (TWR), often also known as a breeder-burner wave reactor, was proposed and developed to address the need for green sustainability, inherent safety, and nuclear nonproliferation in nuclear energy development.^2,3^ TWRs are named for their continuously moving neutron “burning” zone, which continuously consumes new fuel zones and creates a spent fuel zone after burning.^4,5^ As a result, the spent fuel is significantly reduced, thus greatly improving the neutron economy and thus achieving high conversion and fuel consumption ratio characteristics.^6–8^ It is also possible to optimize the design of the TWR reactor using different fuels, including natural uranium, depleted uranium, spent fuel, low-enriched uranium, and even thorium based on different needs.^9^ TWR has outstanding advantages:^10^ ultrahigh nuclear fuel utilization, up to 50–70 folds that of the current light water reactor fleet, high system thermal efficiency, simple control and long-term self-stabilizing operation, and ultralong lifetime extending to about four times or above of the current limit set for fast neutron reactors by IAEA, etc. Once proposed, it was widely regarded as the ultimate form of fission nuclear reactors. Once realized, the TWR technology will disruptively break the current contradiction between safety and economy faced by existing Generation III and IV reactors.

Proposed TWR concepts usually adopt metallic nuclear fuel designs due to their very high breeding ratio among various nuclear fuel forms. Metallic fuels also possess extraordinary inherent safety features that have been demonstrated in real reactor loss of flow accident (LOFA) and loss of heat sink accident (LOHSA).^11,12^ In this article, we propose a novel metallic fuel concept based on the rationale of Chinese “Tai-Chi” Kungfu element, which essentially maneuvers unconventional reactor operations to mitigate performance challenges of fuels and materials faced by the TWR fuel design. In the following, the challenges to the fuels and cladding materials designs are outlined first. The disadvantages of a conventional approach to design TWR fuels are analyzed before the novel design concept is described in detail.

## Challenges on fuels and cladding materials toward high burnups

### High swelling, high gas release, and high FCMI

Despite its obvious advantages on thermal conductivity, breeding ratio, and inherent safety, metallic fuel has some obvious disadvantages. On one hand, it is easy for metallic fuels to form phases between cladding and fuel slug with low solidus temperature; on the other hand, metallic fuels are subject to excessive fuel swelling caused by fission gas bubbles and voids under typical fast reactor conditions. Irradiation-induced swelling, and fission gas release are key limiting factors that restrict fuel performance of metallic fuels toward high burnup. To be specific, U-10Zr and U-Pu-10Zr fuels experience rapid swelling during the first several fractional percent FIMA (fissions per initial metal atom) up to 2% FIMA burnup until the fuel volumetric swelling strain reaches a threshold. At this threshold of fuel swelling strain (typically 30% volumetric strain), a large fraction of fission gas in the metallic fuel is vented to the gas plenum by a fission gas release mechanism related to formation and interconnection of open porosity.^13^ Thus, once the fuel swelling threshold is reached, fission gas can only make a marginal contribution to the extra swelling strain in the mid fuel burnup range until open porosity becomes closed by accumulation of solid fission products. The prominent volumetric increase of metallic fuels needs to be accommodated in order to relieve fuel cladding mechanical interaction (FCMI). Fission gas, which either stays in the fuel matrix, causing significant swelling, or gets vented to the gas plenum, leading to high internal gas pressure, poses a major challenge to the fuel design.^14^

### High FCCI

Fuel cladding chemical interaction (FCCI) has been considered the most important life-limiting factor of metallic fuels at mid- to high burnups. The rapid fuel swelling at 1–2% burnup brings the fuel and the cladding into contact, and the interdiffusion of fuel and cladding constituents forms eutectic phases near the fuel-cladding interface.^15^ For instance, U_6_Fe and UFe_2_ can be formed by the interdiffusion of uranium and iron.^16^ These eutectic phases can potentially lead to fuel melting during reactor transients.

A more important aspect of FCCI is related to the infiltration of lanthanides (mostly Nd and Ce) into the cladding that causes localized embrittlement of the cladding. This is a complicated process as the regions near the fuel-cladding interface are actually composed of several distinct FCCI layers. In addition to lanthanide precipitates along prior austenite grain boundaries, compounds composed of lanthanides and cladding components were also found in previous studies. Some of the compounds were determined to be (Fe,Cr)_17_Ln_2_ or (Fe,Cr)_3_Ln, and the remaining are yet to be determined. The cladding regions penetrated by lanthanides have little mechanical strength and are termed “wastage zones” in previous FCCI studies.^16,17^ It is currently believed that the lanthanides are transported into the cladding through a “liquid-like” diffusion mechanism.^16^ The interconnecting pores in irradiated metallic fuels are filled (or at least partially filled) with liquid sodium from the initial sodium bond and volatile elements such as cesium from fission reactions. These liquid-filled pores offer accelerated diffusion paths for lanthanides. Although volatile fission products could not be avoided, elimination of liquid sodium (or other liquid metals) has the potential to significantly suppress such “liquid-like” diffusion processes and delay the onset of lanthanide attack near the inner surface of the cladding.

### High fast neutron irradiation dose

Ferritic/martensitic steels such as HT-9 and 9Cr steels are being recognized as candidate materials for the metallic fuel-cladding in SFRs due to their high thermal conductivities, low expansion coefficients, and superior irradiation resistances to void swelling.^18^ When materials are subject to the extreme conditions in a reactor, the irradiation-induced supersaturated point defects and clusters are inevitably affected by the coupling effect of stress and temperature, resulting in radiation damages and mechanical degradation of materials. The irradiation creep, which occurs under tensile stress lower than the yield stress, has been found to seriously affect the lifetime of materials, including fuel-cladding. This is also a major challenge for metallic fuels at high fuel burnup.

### Long-term corrosion of cladding materials

Liquid metal (e.g., lead or lead–bismuth eutectic [LBE]) is used as a coolant for advanced fast reactors due to their excellent heat transfer properties, such as low melting point, high thermal conductivity, low viscosity, etc. However, liquid metal is naturally highly corrosive, especially at high temperatures. In the fast reactor environments, corrosion of the metallic fuel-cladding materials occurs primarily through the selective dissolution of alloy components into the liquid metal coolants, and the corrosion rate depends on the composition of the alloys and impurities of the liquid metal.^19,20^ Moreover, the liquid metal could penetrate into the cladding materials along the grain boundaries and induce intergranular corrosion.^21^ The composition and structural changes due to the selective dissolution and intergranular corrosion will lead to metallic cladding materials failure, especially under the high-temperature flowing lead or LBE conditions.

In view of the corrosion and degradation of metallic materials in lead or LBE environments, a widely used way of mitigating corrosion is to form a protective oxide scale on the surface of metallic materials by controlling the oxygen concentration that allows oxidation of the alloy but not oxidation of the liquid metal. For temperatures above 500°C, other measures such as coating or surface alloying need to be taken because the consumption of alloys due to oxidation could be rather fast at higher temperatures. Although these methods can mitigate materials corrosion, they are insufficient for protecting metallic cladding at high fuel burnup. It is found that the protection of alloys by forming an oxide layer has high requirements on oxygen control, and the oxide scale could fail due to the dissolution or thermal stress during the long-term exposure in liquid metal. Similarly, the disadvantage of material surface coating or surface alloying is that it is not self-repairing, and has the risk of cracking and peeling off due to thermal stress induced by different coefficients of thermal expansion compounded by the rather strong irradiation effects at high fuel burnups. Corrosion of cladding materials is another major challenge for the high fuel burnup conditions.

## Conventional approach to the materials challenges

The conventional approach to face the fierce materials challenges is to utilize various design elements to minimize the damage caused by each of the previously mentioned threats and extend materials tolerance to these severe conditions as much as possible. For example, ODS steels have been proposed, which is expected to offer better performance characteristics under high-temperature,^22,23^ high corrosive environments,^24^ and high irradiation dose.^25,26^ The underlying mechanism to support such performance improvement relies on the strengthening and enhanced defect sink strength by the dispersion oxide particles.^27^ In addition, several metallic fuel designs were adapted to resolve the challenges posed by swelling and fission gas release under ultrahigh burnup: a low SD (smeared density), as low as 55%, was employed to accommodate the rapid swelling and avoid premature mechanical failure of the cladding; a gas venting mechanism to relieve the stress induced by the large amount of released fission gas; coating or liner on the cladding inner surface and/or targeted fuel alloy additions or adopting the annular metallic fuel design to mitigate FCCI. However, whether it's on the fuels or cladding materials, the conventional approach seems to only be able to further extend the failure limits by margins not satisfactory enough to meet the design requirements of TWRs.

Moreover, the efforts on the development of fuels and cladding materials more or less suffer from a scattered state: fuel performance analyses, analyses of irradiation effects and damage, corrosion analyses, and analyses of chemical processes were performed individually. A more integral approach has not yet been well established. For example, cladding materials such as HT-9 steels have been put to irradiation tests greater than 200 dpa, but the effects of a realistic stress state on the cladding tube and the synergistic effects were not considered.^28^

## The novel “Tai-Chi” approach

In this novel approach we call “Tai-Chi” approach, we present a fresh viewpoint with which we aim to gain at the seemingly unreachable 600 dpa irradiation dose limit on the cladding materials, higher than 40% FIMA burnup target on the fuels, 40–60 years of extensive corrosion time length on the cladding materials, at the same time. The reason behind the use of the word “Tai-Chi” is that the core philosophy of mitigating the fierce challenges by utilizing the possible benefits inherent to these challenges themselves is in good coherence with the Chinese “Tai-Chi” Kungfu element.

**Figure 1:**
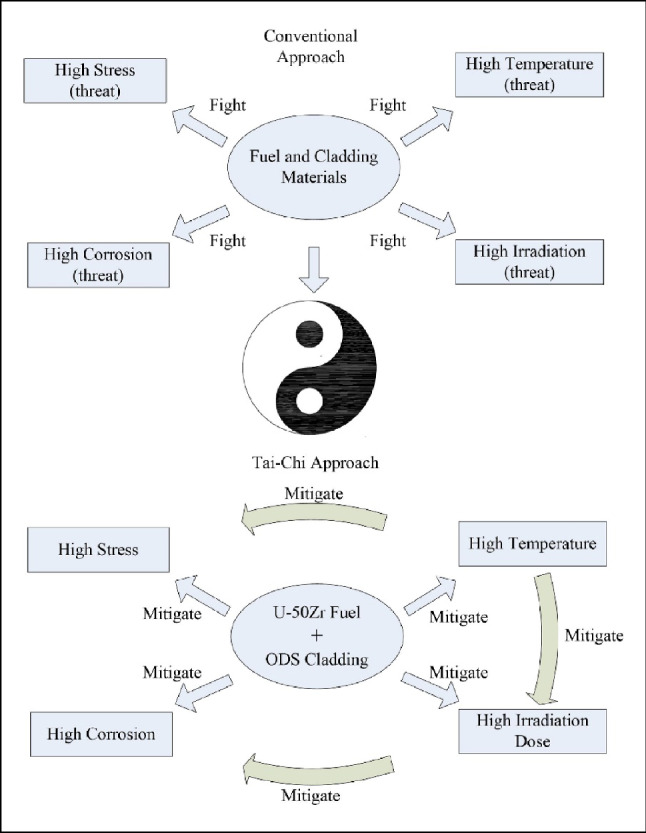
Schematic of the shift from a conventional approach to a novel “Tai-Chi” approach in solving the traveling wave reactor ultrahigh burnup metallic fuel problems. ODS, oxidation dispersion strengthened.

The schematic of moving from the conventional approach to the novel “Tai-Chi” approach is laid out in **Figure** **1**.

## Detailed approach on “mitigating” the challenges

### Use of annular U-50Zr fuel, vented gas to mitigate the issue of high stress and FCCI

In order to reach a burnup as high as 40% FIMA or even higher, the FCMI issue is the most serious challenge as it becomes the biggest contributor to cladding failure after about 10–12% FIMA burnup for 72–75% SD U-10Zr and U–Pu-10Zr fuels.^29^ Therefore, in the extensive irradiation length of the fuel under steady-state operation conditions, the fuel has to be swelling-resistant itself in the first place. FCCI is believed to be another major contributor to cladding failure, particularly under high temperatures. The fuel design has to inhibit the high-temperature eutectic formation scenario throughout its life cycle. The U-50Zr fuel possesses very peculiar and desirable characteristics viewing from these angles: it undergoes a spinodal decomposition transitioning from a hexagonal crystal structure phase to two separate cubic crystal structure phases, which are essentially swelling-resistant; it has a high-Zr content that diffusion processes are suppressed, which mitigate both the constituent redistribution issues and the FCCI issue of fission products corroding the inner surface of cladding materials;^30^ at high enough temperature, the fuel evolves into a high plasticity γ-phase that can effectively creep toward the hollow center under an annular design upon fuel-cladding contact to reduce FCMI; the phase transition plus high temperature will promote extensive fission gas release, which will render the fuel material highly porous and hence FCMI ineffective.

An annular fuel design not only serves the purpose of allowing inward swelling at high-temperature scenarios and thereby reducing FCMI, but also acts as a way to alleviate FCCI issues. In the annular fuel design, the gap between the fuel and the cladding is significantly reduced compared to the solid (rod) design, allowing the use of inert helium gas as the heat transfer agent. The poor thermal conductivity of helium gas can be compensated by the small fuel-cladding gap. This eliminates the necessity of using liquid metal as the bonding material. Therefore, the annular fuel design can provide significant back-end benefits for fuel recycling.^31,32^ Researchers have reported that in an annular U-10Zr fuel test, lanthanides did not accumulate at the fuel surface as they did in solid U-10Zr fuels.^33^

Even for U-50Zr fuel, it is impossible to prevent fission gas from getting released to the gas plenum in large quantities. It needs to be stressed that fission gas release tends to be very strong in metallic fuels. Consequently, a vented fuel design was in place for the ultrahigh burnup metallic fuel design concept proposed by ANL researchers. Although fission gas release is considered less severe in low diffusion metallic fuel such as U-50Zr, it is ungrounded to think that fission gas release will still be suppressed at a burnup level as high as 40% FIMA. After all, any temperature surge event would fundamentally change the gas equation of state so as to promote gas release. As such, rather than attempting to contain the generated fission gas, the fuel design proposed in this work adopts the idea of vented fuel in the earlier ANL design.

In this design, fission gas release is in fact welcomed as any fission gas contained in the fuel will eventually form bubbles and cause fuel swelling to some extent. Thus, it becomes imperative to release nearly all gas generated in the fuel. This is obviously another challenge. In this work, the most important key design element is actually to purposefully introduce a high-temperature transient, though well controlled and safe so it could be viewed as a “normal” reactor operation. There is a common misunderstanding in metallic fuels, that is, its low melting temperature and hence the small temperature difference between its operating temperature and the solidus line is a huge disadvantage of the fuel. However, one needs to consider not only the magnitude of the temperature difference but also the magnitude of temperature rise per unit input power. The U-10Zr metallic fuel is fabulous in its ability to resist power and temperature surge scenarios. The LOFA and LOHSA experiments performed on the EBRII reactor completely astonished the entire world as a perfect showcase of the inherent safety feature of metallic fuels.^11,12^ The more recent experiments by Kilopower has demonstrated the perfect load following abilities of yet another metallic fuel form, UMo.^34^ The strong negative temperature feedback largely relaxes concerns with such fuel operating at high temperatures over a short period of time.

**Figure 2:**
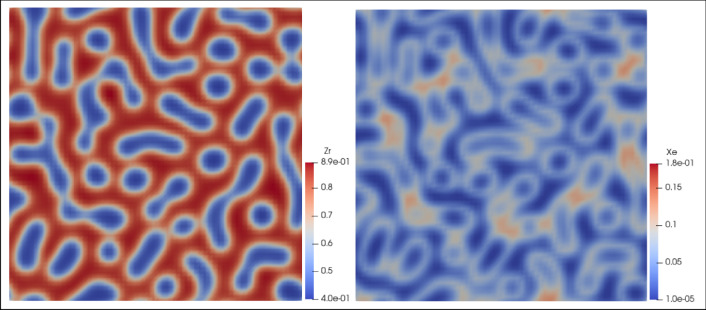
(Left) The distribution of Zr-rich phase (red zones) and U-rich phase (blue zones) and (right) the distribution of fission gas atoms in U-50Zr in a spinodal decomposed state (~4% fissions per metal atom burnup).

**Figure 3:**
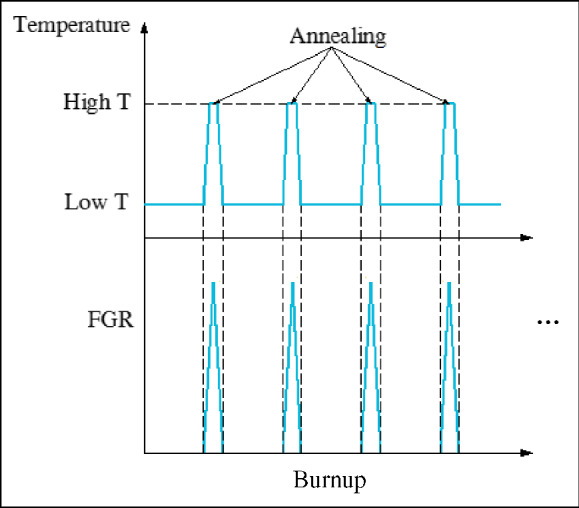
Schematic of fission gas release (FGR) with cyclic high-temperature transient scenarios.

In fact, we purposefully introduce a high-temperature transient operation scenario, which is aimed at releasing all stored fission gas so as to prohibit strong swelling due to existing fission gas contained in the fuel matrix once the fuel is back in its lower temperature steady-state operation. Phase field simulations (the details of which are provided in the Supplementary Materials) showed that fission gas atoms and bubbles tend to accumulate on the phase boundaries between the two cubic phases, as depicted in **Figure** **2**, which is a location that is believed to foster gas release. When fuel temperature is raised above the phase-transition temperature of U-50Zr fuel (630°C, but this temperature tends to shift to a higher value when irradiation effects are present), the phase-change process will bring about significant driving force for gas atom diffusion. In addition, when the temperature reaches a higher temperature exceeding the phase transition point, fission gas will be driven to be released due to the high temperature. It is reasonable to expect that fission gas release will be near 100% once fuel temperature exceeds 850°C. This is consistent with the commonly observed fission gas release behaviors in fast reactor fuels such as those in UN, that gas release would be nearly full when temperature exceeds 50–55% of the fuel melting temperature.^35^ In such a way, a cyclic operation procedure may be established that accumulates fission gas in the fuel matrix on phase boundaries during steady-state operation for the long-term, and then releases a large fraction of these gases during the high-temperature transients over a short-term operation. A schematic of cyclic fission gas release is provided in **Figure** **3**. Venting fission gas in this way can actually help gauge the real-time fuel temperature in this transient process through pressure sensors in the gas vent line in a fuel assembly. The readily available 3D printing technology renders design of complex geometry thin-walled pipelines feasible for such a gas collection and pressure measurement system.^36^ Work is in progress to carry out an exact design of this gas collection system with pressure sensors and an algorithm to back out the temperature distribution in the fuel at real time with a good precision level.

Finally, when the high-temperature transient operation is over, temperature of the fuel is gradually taken down to the steady-state operation temperature where a phase transition from the high-temperature γ-phase back to the lower temperature spinodal decomposed cubic phases takes place. The phase transitions from the lower steady-state temperature to the higher transient counterpart and then back to the original state will help sweep the accumulated irradiation defects from within the fuel matrix to the phase and grain boundaries. As fission products can lead to FCCI issues when they migrate to the fuel outer surface, the behaviors of these fission products under phase transitions and high-temperature operations need to be clarified in the future. The annular fuel is, in fact, specifically designed to keep a gap between the fuel slug and the cladding over steady-state long-term operation, so as to prevent FCCI by accumulation of lanthanide fission products at the outer surface of the fuel.

### Utilizing irradiation-enhanced diffusion to mitigate the corrosion problem

Ferritic/martensitic steels such as HT-9 and 9Cr steels are being recognized as candidate materials for the metallic fuel-cladding in fast reactors.^37^ It is known that ferritic/martensitic steels derive their corrosion resistance from the formation of compact oxide scale on the surface of materials by carefully controlling the oxygen content in the liquid metal.^38^ It has been reported that the oxide layer has a duplex structure composed of a compact Fe–Cr spinel inner layer and a porous external magnetite layer in contact with the liquid metal.^39^ According to the “available space model,”^40^ on one hand, iron ions diffuse from the metal to the external interface leading to the growth of magnetite and formation of vacancies that could accumulate to form nanocavities at the metal/Fe–Cr spinel interface. On the other hand, the Fe–Cr spinel layer grows at the metal/oxide interface because oxygen diffuses easily inside the oxide scale through the nanocavities until the internal interface to generate Fe and Cr spinel. It is the outward diffusion rate of Fe rather than the inward diffusion rate of oxygen that controls the corrosion rate of ferritic/martensitic steels.^41^ Thus, an effective method to reduce the corrosion rate of cladding materials is to reduce the diffusion rate of Fe through Fe–Cr spinel oxide.

**Figure 4:**
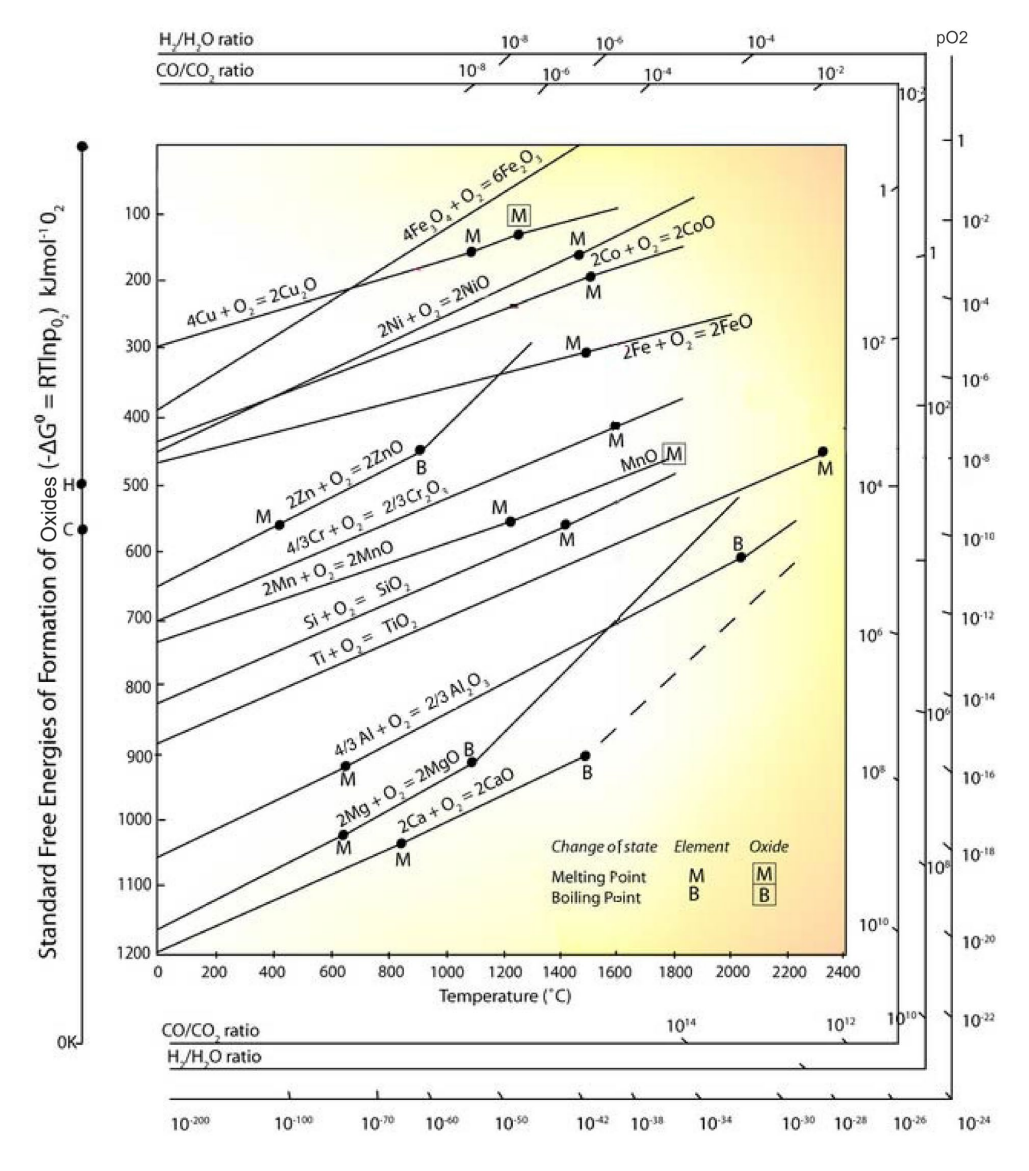
Ellingham diagram for metal oxides.^42^

An Ellingham diagram of Gibbs fee energy change versus temperature for the formation of metal oxides is shown in **Figure** **4**. Based on the thermodynamic calculations, it is seen that the formation Gibbs free energies of Cr, Mn, Al, and Si oxides is lower than that of Fe, indicating that the addition of Cr, Mn, Al, and Si contents in cladding materials is beneficial to enhance the oxide scale compactness and therefore can reduce the outward diffusion of Fe and improve its corrosion resistance in liquid metal environments.^42^ However, due to the lower contents of Mn, Al, and Si in ferritic/martensitic steels (e.g., the Mn content is usually lower than 1 wt%), the oxide scales formed on their surface are Fe–Cr oxides. Thus, if we could find a method, without changing the composition of the ferritic/martensitic alloys, which can effectively enhance the diffusion rate of Mn and Cr and increase the concentration of these elements at the oxide layer, it will significantly improve the corrosion resistance of cladding materials in liquid metal and could be satisfactory enough to meet the design requirements of TWRs.

**Figure 5:**
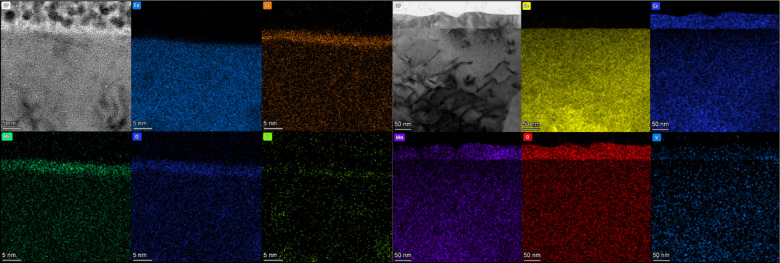
Cross-sectional image and corresponding energy-dispersive spectroscopy maps of MX-ODS steel without (left) and with (right) Fe irradiation at 550°C.

It has been reported that the composition and structure of oxides on materials are different after different surface treatment. Chen et al*.*^43^ studied high-temperature oxidation characteristics of ultrafine ferrite—martensitic steel in air at 650°C and its corrosion behavior in liquid LBE. Results showed that a slight improvement in oxidation resistance was observed in an ultrafine-grained sample fabricated by 94% cold forge deformation. It is known that cold forge could enhance the high-angle grain boundaries as well as defects and dislocation concentrations, and accelerate the diffusion rate of Mn.^44^ Therefore, a Mn-rich oxide (MnCr_2_O_4_ and Mn_2_O_3_) was formed in the ultrafine-grained ferritic/martensitic steels and the presence of Mn-rich oxide suppressed the corrosive attack of LBE and the outward diffusion of Fe. Similarly, a large amount of vacancies and interstitials can be produced when materials are subjected to irradiation, especially upon high-dose fast neutron irradiation. The high defect concentrations could accelerate the diffusion and improve the oxidation behavior of materials. Yao et al*.*^45^ reported that ion irradiation not only resulted in a significant increase in thickness of surface oxides, but also remarkably modified the microstructure of oxides in comparison with the corroded samples without irradiation. The effect of irradiation on the corrosion process was believed to be mostly related to the radiation-enhanced diffusion. We found similar results in our recent studies.** Figure** **5** presents the oxide scale formed on the surface of MX-ODS steel without and with Fe irradiation at 550°C (sample without irradiation was an area blocked from irradiation but experienced the same 550°C conditions). As shown in Figure 5, a compact oxide film with an average thickness of about 40 nm was formed on the surface of ODS steel after Fe irradiation, which is much thicker than that without irradiation (the oxide film without irradiation was around 5 nm according to Figure 5). In addition, the oxides are rich in Mn and Cr according to the EDS maps. Combined with XRD and TEM, it was revealed that the oxide film formed on the MX-ODS steel was (Cr, Mn)_2_O_3_ after irradiation. The irradiation-induced supersaturated point defects and clusters are inevitable in advanced fast neutron reactors or TWR. Based on the previously discussed results, the extreme radiation environments in reactors can possibly provide a potential way to accelerate the diffusion of elements and improve the thickness and structure of oxides formed on the candidate cladding materials and replenish irradiation-induced defects, such as nanovoids and nanoscale cracks, by supply of additional matrix atoms, before the defects further grow to become extensive. In fact, recent research has reported that proton irradiation decelerates intergranular corrosion of Ni–Cr alloys in molten fluoride salt at 650°C. Researchers demonstrated this by showing that the depth of intergranular voids resulting from Cr leaching into the salt is reduced by proton irradiation. Irradiation-induced interstitial defects enhanced diffusion, more rapidly replenishing corrosion-induced vacancies with alloy constituents, thus playing a crucial role in decelerating corrosion.^46^ Thus, the corrosion resistance of the metallic cladding in liquid metal environments can be improved.

It should be pointed out that although irradiation could improve the oxide structure and thickness of oxide scale, the irradiation-accelerated corrosion would be a challenge as well for the development of metallic cladding materials. It is known that the solubility of Mn in pure lead is much higher than that of Fe and Cr, that could affect the long-term corrosion resistance of Mn-rich oxide in liquid metal. Thus, in the future, more irradiation-oxidation and irradiation-corrosion experiments will be necessary to confirm the feasibility of utilizing irradiation-enhanced diffusion to treat the corrosion problem of metallic cladding in advanced fast neutron reactors or TWRs.

### Use of controlled high-temperature transient to anneal cladding materials in-pile

It has been long believed that there will be so many problems, which the cladding materials need to face in order to survive 600 dpa or higher fast neutron irradiation dose. The issue of irradiation-induced breakaway void swelling has to be solved in the first place. The best performing swelling-resistant material that has been evaluated thoroughly is the ferritic/martensitic HT-9 steel, which has the potential to perform well up to about 200 dpa.^47^ In view of the vast gap between the do-able and the desirable, we hereby propose an unorthodox approach of “mitigating” the swelling issue by utilizing the benefits of the earlier proposed high-temperature transient (i.e., in-pile heat treatment [or thermal annealing]).

Swelling is known to have an incubation period when voids stay at small sizes after nucleation and when vacancy supersaturation has not been fully established.^48^ This incubation period could extend from 30 to 40 dpa in austenitic steels to more than 80 dpa in F/M steels such as HT-9.^18^ It is believed that such incubation period could be even longer for ODS steels. A large amount of irradiation tests have demonstrated that swelling has a bell shape dependence on temperature and at high enough temperatures irradiation swelling will remain low due to activation of diffusion of defect clusters of opposite natures (i.e., interstitial and vacancy), which then leads to annealing of irradiation defects.^49^ In our scenario, the situation is slightly different as it acts more like an out-of-pile annealing when the defects are generated by long-term irradiation effects at lower temperatures (i.e., steady-state operation) while they are annealed at a higher temperature for a comparatively much shorter time length (6–8 h), as schematically illustrated in Figure 3. However, as long as the vacancy supersaturation has not been established, raising the cladding temperature will result in annealing effects. The reason that in-pile annealing has not been done conventionally is that, on one hand, the cladding may undergo plastic deformation at such high temperatures; on the other hand, even if the cladding remains elastic, the creep would become so high that the cladding could breech in hours, if not minutes. In such a case, it is not that there cannot be a high stress level on the cladding materials, it is rather that there cannot be nearly any stress to activate significant thermal creep. As we suggested earlier, FCMI could be effectively mitigated once the fuel operates at a high enough temperature in its γ-phase. This has been, in fact, demonstrated in the AFC-3 rodlet tests when abnormal high temperature was induced due to manufacturing problems in the fuel-cladding interface.^31^ But then, even if FCMI can be maintained at a low level, it does not necessarily mean it is low enough to avoid the thermal creep scenario.

**Figure 6:**
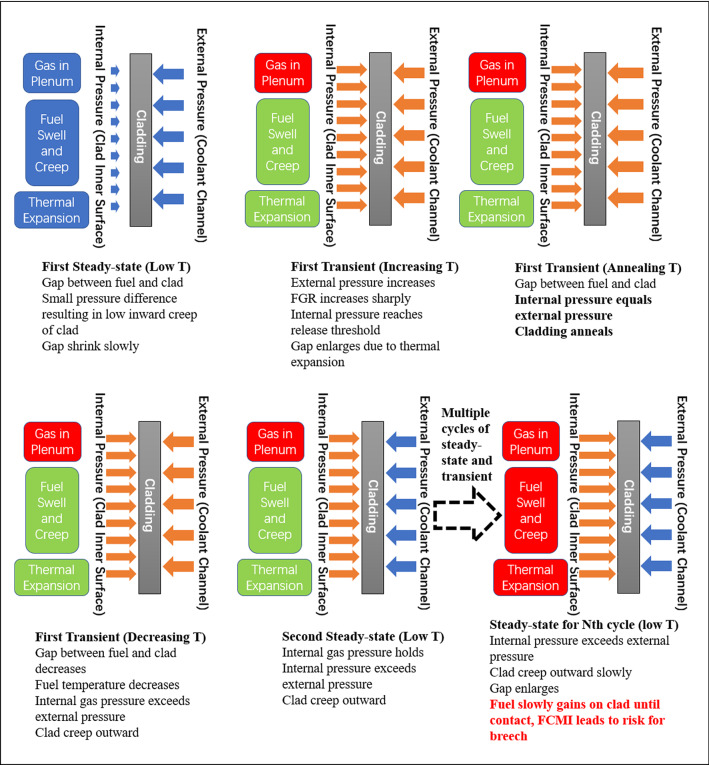
Relationship between gas pressure in plenum and pressure in the coolant channel over multiple cycles of steady-state and transient operations (Box colors: blue: initial pressure level; green: low pressure level; red: high pressure level; arrow colors: blue: initial pressure level; brown: pressure at elevated levels).

Therefore, in this design, we introduce another key element: a force balance mechanism on the cladding material under the high-temperature transient operation scenario. We begin by introducing a pressure in the primary loop or the pressure vessel for the pool design and introduce an internal pressure in the fuel pin with prefilled He. The internal pressure is purposefully set slightly lower than the external one, and the small pressure difference and the net external pressure are not going to cause issues under the steady-state operation as the temperature of the cladding does not allow effective creep. In addition, the strength of MX-ODS or 14YWT ODS steels at the steady-state operation temperature regime is robust from a design perspective.^50,51^ The U-50Zr fuel is designed to be annular in shape leaving a gap between the fuel and the clad to accommodate fuel outward swelling during the steady-state operation.^52^ The design of the central hole of the fuel is a little intricate as it serves two main purposes: (1) due to the axial temperature difference between the bottom part and top part of the fuel pin, the top higher temperature part will see phase transition even at the low temperature steady-state operation, the central hole then leaves space for the high-temperature γ-phase to creep inward due to the stiff nature of the outer spinodal decomposed cubic phase nanostructured U-50Zr fuel; (2) one would also want the fuel central hole to close at very high burnup so the fuel only experiences a gas pressure from the gap to press it so as to further inhibit strong swelling. On raising the cladding temperature, the external pressure on the cladding will increase according to the ideal gas law. As the coefficients of thermal expansion of the clad are higher than that of the fuel, the gap between the fuel and clad will enlarge.^18,53^ The resulting fuel temperature rise due to gap enlargement and temperature rise of the coolant will produce one major effect: large amounts of fission gas will be released to the plenum due to the temperature rise. An internal pressure control mechanism is proposed here, which relies on a pressure control device at the top fuel cap designed by researchers from ANL.^54^ A diving bell type device is put in place so that only when fuel internal pressure exceeds a certain level does it open to vent the gas. Once enough gases are vented to the gas collection system, the pressure reduces back to the pressure control level set by the mass of the diving bell such that the internal pressure in the plenum can be effectively maintained at a constant. At high temperatures, the fuel, after releasing fission gas, will be a porous material, and its high creep rate will relieve FCMI even if the fuel contacts the clad. And the internal gas pressure is effectively operating on the internal surface of the cladding material, so a very intricate force balance may be obtained if the internal control pressure is set to be exactly the same as the expected pressure in the coolant channel at the designated cladding heat treatment temperature. A schematic of such a design in shown in **Figure** **6**. Under such a force balance, the strain increment of the MX-ODS or 14YWT ODS steels can be kept at a low level, if not none, such that cladding failure due to significant thermal creep is avoided. The irradiation effects in the MX-ODS or 14YWT ODS steel cladding materials are left to annealing at the high temperature for the transient operation for 6–8 h.

The detailed cycle-by-cycle operation and the relationship between gas pressure in plenum and pressure in the coolant channel is illustrated in Figure 6. It is further elaborated that if the pressure by the diving bell can be controlled by any means, a more straightforward mechanical balancing scheme may be achieved by setting the plenum inner pressure to the outer pressure in the primary loop.

It needs to be further noted that the axial temperature gradient on the cladding requires us to consider both the higher temperature top part and the lower temperature bottom part of the cladding at these cyclic operation cycles. In order to avoid significant axial stress gradient, the reactor is proposed to run at zero power during the high-temperature annealing operation generating only decay heat. The heat exchanger interfacing the primary and secondary circuits will be operating at a low heat sink rate such that temperature of the primary coolant will rise slowly and flatten off at a designated temperature for annealing of cladding materials.

Moreover, when a fast reactor operates to very high fuel burnup levels, large amounts of helium will be produced forming helium bubbles. Formation of large helium bubbles tends to foster swelling and embrittlement of cladding. However, it has been demonstrated that in high-performance ODS steels such as 14YWT, helium bubbles remained small and dispersed after high-temperature annealing at 900°C in specimens pre-implanted with about 12,000 appm peak helium concentration, proving that helium bubble growth can be well suppressed in this ODS steel even at such high temperature.^55^ As such, thermal annealing at temperatures not higher than 800°C for 6–8 h in our design will very probably not cause significant helium bubble growth nor the resulting high swelling.

Finally, it needs to be stressed that other aspects such as neutronics and thermohydraulics of this fuel design are out of the scope of this work and therefore their discussions are not included in this article. U-50Zr, due to the low uranium loading, may have issues in sustaining the traveling wave so a special core design or a mixed use of high-Zr and low-Zr fuels may be necessary.

## Summary

In this article, we carried out a fuel element design at a conceptual level to serve the purpose of reaching ultrahigh fuel burnup for a metallic fuel form. The core disadvantages of the conventional approach were laid out where the raised challenges are believed to be so fundamental and profound that such conventional approach is rendered ineffective. Based on the rationale of the Chinese “Tai-Chi” Kungfu element, a new and unorthodox approach was proposed to utilize the possible benefits of high temperature to manifest periodic fission gas release to relieve the FCMI challenge and, at the same time, use the annealing benefits of such high-temperature operation to treat irradiation effects on the cladding materials. In addition, a phenomenon that irradiation boosts transport of Mn and Cr elements to the cladding surface to form a dense and protective thin oxide as a self-repairing mechanism was discovered and is believed to be able to help mitigate the long-term corrosion challenge by liquid lead. This innovative conceptual design shifts the conventional strategy to maximize materials irradiation tolerance from a sole materials perspective to a new strategy that maneuvers thermohydraulic operations to elongate materials life under the extreme conditions in a TWR-type fast reactor.

## Supplementary Information

Below is the link to the electronic supplementary material.

Supplementary file1 (DOCX 94 KB)

## Data Availability

Data of this work will be made available upon reasonable request.
