# Supplementary material for: A novel approach on designing ultrahigh burnup metallic TWR fuels: Upsetting the current technological limits
Source: MRS Bull. 2022 Nov 3;47(11):1092–102. doi: 10.1557/s43577-022-00420-4 (PMC9632587; doi:10.1557/s43577-022-00420-4)
Supplement: Supplementary file 1 — Supplementary file1 (DOCX 94 KB) [file 43577_2022_420_MOESM1_ESM.docx]

**Details of the phase field simulations (results are presented in Fig. 2)**

We began by adding the effects of fission gas into the phase-field model of spinodal decomposition. The effect of fission gas on spinodal decomposition derives from bubbles and gas atoms. In our model, we regard bubbles and matrix as two phases, expressed by the order parameter$\eta$. It takes the value of 1 within bubbles, and drops to 0 outside the bubbles and varies smoothly through the phase interface. Gas atoms are both in bubbles and matrix. In the system where spinodal decomposition occurs, we set the concentration of the component Zr as$c_{A}$and the concentration of gas atoms as$c_{g}$. They are treated as conservative variables evolving over time. The defect effects were also considered in our model. The vacancy concentration$c_{v}$was introduced and we took it as a constant for simplification.

In the thermodynamic phase models, the total free energy, $F$, consisting of the bulk free energy and interfacial energy, of a system with inhomogeneities in both concentrations and parameter order is written as a volume integral：

$$F=\int_{V} \left\{\frac{1}{V_{m}}[(1-h(\eta))f_{m}+h(\eta)f_{b}]+wg+\frac{1}{2}\kappa_{c_{A}}(\nabla c_{A})^{2}+\frac{1}{2}\kappa_{c_{g}}(\nabla c_{g})^{2}+\frac{1}{2}\kappa_{\eta}(\nabla\eta)^{2}\right\}dV$$

(1)

Where, $V_{m}$ is defined as relative volume , $N_{A}$is Avogadro's constant and$a$is the lattice parameter of U-50Zr. $h(\eta)$is an interpolation function of $\eta$, $h(\eta)=\eta^{3}(6\eta^{2}-15\eta+10)$,$g$is a barrier function, expressed as$g=\eta^{2}(1-\eta)^{2}$. $w$is the height of the double potential well function; $\kappa_{c_{A}}$is the concentration gradient energy of coefficient component Zr; $\kappa_{\eta}$is the energy coefficient of the phase fraction gradient; $w$,$\kappa_{\eta}$determine the shape and width of the phase diffusion interface. $f_{m}$ is the matrix free energy and $f_{b}$ is the bubble phase free energy. $f_{m}$ can be expressed using solid solution model.

We consider using the traditional double-well potential function and parabolic function to describe the free energy [1,2].

$$f_{m}=A(0.4-c_{A})^{2}(0.9-c_{A})^{2}+B(c_{v}-c_{v}^{eq})^{2}+C(c_{g}-c_{g}^{eq})^{2}$$

$$f_{b}=D(c_{v}^{b})^{2}+Ec_{v}^{b}+F+G(c_{g}^{b})^{2}+Hc_{g}^{b}+I$$

where the parameters from $A$ to $I$ are set as constant simply. $c_{j}^{eq}$ is the thermal equilibrium concentration of $j$ with $c_{j}^{eq}=\exp\left(-\frac{E_{j}^{f}}{k_{B}T}\right)$, $j=v,g$. Here we set the value of $E_{j}^{f}$as 3eV and temperature as 550℃ in our simulation according to the simulated conditions.

The evolution of the composition field $c_{A}$and $c_{g}$ are governed by the Cahn–Hilliard equation for conserved variables

| $\frac{\partial c_{A}}{\partial t}=\nabla\cdot(M_{c}\nabla\frac{\delta F}{\delta c_{A}})$ | (4) |
| --- | --- |
| $\frac{\partial c_{g}}{\partial t}=\nabla\cdot(M_{g}\nabla\frac{\delta F}{\delta c_{g}})$ | (5) |

The KKS model [3] was used here to deal with the relationship between matrix, bubbles and gas atoms. Following the KKS model, the concentration of gas atoms is written as:

| $c_{g}=(1-h)c_{g}^{m}+hc_{g}^{b}$ | (6) |
| --- | --- |

The KKS formalism also requires the chemical potentials of coexisting phases to be equal, and it should be noted that the equality does not take place only in the interfacial region.

| $\frac{\partial f_{m}}{\partial c_{g}^{m}}=\frac{\partial f_{b}}{\partial c_{g}^{b}}$ | (7) |
| --- | --- |

It is reasonable to assume that there are no component atoms in the bubbles, that is, the components only exist in the matrix. $\frac{\delta F}{\delta c_{A}}$ and $\frac{\delta F}{\delta c_{g}}$are the chemical potentials whose gradient drives the diffusion of component A and gas atoms, respectively. $M_{c}$ and $M_{g}$are the atomic mobility of component A and gas atoms, respectively. $M_{g}$is expressed by atomic mobility of both matrix phase and bubble phase.

| $M_{g}=(1-h)M_{g}^{m}+hM_{g}^{b}$ | (8) | |
| --- | --- | --- |
| $M_{g}^{i}=\frac{D_{0}^{i}V_{m}}{RT}\exp\left(\frac{-Q_{i}}{RT}\right)$, with $i=m,b$ | | (9) |

where $D_{0}^{i}$is the pre-exponential factor of the diffusion coefficient [4], and $Q_{i}$[4] is the activation energy of the mutual migration of U and Zr in the i phase (i stands for α, β, and γ).

We used the finite difference method to simulate the spinodal decomposition phenomenon taking into account the interaction of gas atoms and bubbles in U-50Zr. 64 grid points were selected for each length and width and $\Delta x=\Delta y=1$. The time step is 0.01, and the simulation ran for 10000 steps. The simulation results are shown in Fig.2 in the content of the paper.

Reference

[1] Li, Y., Hu, S., Montgomery, R., Gao, F., & Sun, X. (2013). Phase-field simulations of intra-granular fission gas bubble evolution in UO2 under post-irradiation thermal annealing. Nuclear Instruments and Methods in Physics Research Section B: Beam Interactions with Materials and Atoms, 303, 62-67.

[2] Aagesen, L. K., Gao, Y., Schwen, D., & Ahmed, K. (2018). Grand-potential-based phase-field model for multiple phases, grains, and chemical components. Physical Review E, 98(2), 023309.

[3] Kim, S. G., Kim, W. T., & Suzuki, T. (1999). Phase-field model for binary alloys. Physical review e, 60(6), 7186.

[4] Hofman, G. L., Hayes, S. L., Petri, M. C. (1996). Temperature gradient driven constituent redistribution in U- Zr alloys. Journal of Nuclear Materials, 227(3), 277-286.
